# Self-learning model fusion for network anomaly detection: A hybrid CNN-LSTM-transformer framework

**DOI:** 10.1371/journal.pone.0332502

**Published:** 2025-10-29

**Authors:** Jun Wang, Ning Huang, Houzhong Zhang, Luyun Liu, Qiang Fu, Kerang Cao, Xiwang Guo, Hoekyung Jung

**Affiliations:** 1 College of Computer Science and Technology, Shenyang University of Chemical Technology, Shenyang, Liaoning, China; 2 Key Laboratory of Intelligent Technology of Chemical Process Industry in Liaoning Province, Shenyang, China; 3 Information and Control Engineering Liaoning Petrochemical University, Fushun, China; 4 Computer Engineering Department, Paichai University, Daejeon, South Korea; University of Calabar, NIGERIA

## Abstract

The rapid evolution of cyber threats poses significant challenges to the adaptability and performance of anomaly detection systems. This study presents an innovative hybrid deep learning framework that integrates Convolutional Neural Networks (CNN), Long Short-Term Memory networks (LSTM), and Transformer models with a novel self-learning mechanism to enhance network traffic anomaly detection. Our key contributions include: (1) a synergistic two-stage model fusion architecture that captures both spatial and temporal traffic patterns; (2) an adaptive learning mechanism with multi-metric drift detection that autonomously responds to evolving threats; and (3) a knowledge preservation strategy that maintains detection capabilities while adapting to new attack patterns. The proposed CNN-LSTM model achieves F1-scores of 0.9778 and 0.9695 on the UNSW-NB15 and CICIDS2017 datasets respectively for binary classification of normal vs. anomalous traffic. The LSTM-Transformer model further classifies specific anomaly types with accuracies of 0.9632 and 0.9528 on these datasets, representing significant improvements over recent methods. Experiments demonstrate the framework’s robustness, maintaining an average accuracy of 0.955 (σ≈ 0.005) over a 15-day simulated period with multiple induced concept drifts. The self-learning mechanism, with multi-metric drift detection and an efficient model update strategy, enables the system to detect drifts and recover performance within 23.4 ± 0.20 hours post-drift, while achieving a 92.8% detection rate for zero-day attacks. The proposed framework offers a promising direction for developing efficient and autonomous cybersecurity systems capable of handling dynamic and evolving threat landscapes.

## Introduction

In the digital age, cybersecurity has become a key issue in protecting national infrastructure, business operations, and personal privacy [1]. With the popularity of Internet of Things (IoT) devices, the promotion of 5G technology, and the widespread application of cloud computing, the complexity of network connections and data traffic have increased exponentially, bringing unprecedented challenges to network security [2]. The contemporary digital landscape has witnessed an unprecedented escalation in the multifaceted nature of cyber threats [3]. This period has been characterized by a marked intensification in the frequency, magnitude, and intricacy of malicious cyber activities. The repercussions of these evolving threats extend beyond mere technological disruptions, manifesting in substantial economic ramifications on a global scale. A comprehensive analysis conducted by Cybersecurity Ventures, a respected authority in the field, projects a staggering trajectory for the financial impact of cybercrime. Their forecast indicates that the aggregate global economic burden attributable to cyber malfeasance is anticipated to reach a monumental $10.5 trillion annually by the year 2025 [4]. This projection underscores the critical imperative for robust cybersecurity measures and highlights the potential for severe economic destabilization in the absence of adequate protective strategies.

Network traffic anomaly detection plays a vital role in the first line of active defense against cyber threats [5]. An effective anomaly detection system can identify known attack patterns and discover unknown threats, providing timely warnings and response opportunities for network administrators [6]. However, traditional anomaly detection methods often exhibit limitations, such as high false positive rates, low detection efficiency, and difficulty in handling zero-day attacks [7,8]. Therefore, the development of advanced and adaptive anomaly detection technology has become a top priority in the field of network security [9].

Early anomaly detection methods relied on statistical analysis and expert systems, with Denning’s statistical model [1] laying the foundation. Machine learning techniques, including support vector machines (SVM), decision trees, and K-nearest neighbors (KNN), emerged in the early 21st century [10], offering improved detection accuracy but facing challenges in feature engineering and generalization.

The landscape of anomaly detection underwent a paradigm shift around 2010, driven by the convergence of big data infrastructures and advanced computational capabilities. Deep learning architectures began demonstrating remarkable efficacy in processing high-dimensional data and identifying complex patterns [11]. This transformation was particularly evident in network security applications, where the need for sophisticated threat detection mechanisms had become increasingly critical.

The evolution of deep learning in network anomaly detection has witnessed several significant developments. Convolutional Neural Networks (CNNs) were first adapted for network traffic analysis by Wang et al. [12], who pioneered the transformation of network data into "image-like" representations. This approach was further enhanced by Ahmad et al. [13], who developed multi-channel architectures capable of processing both raw and statistical features simultaneously. The temporal aspects of network traffic were addressed through Long Short-Term Memory (LSTM) networks, with notable work by Yang et al. [14] integrating bidirectional LSTM with attention mechanisms to capture complex sequential patterns.

Unsupervised learning approaches have also made substantial contributions to the field. Autoencoder-based systems, such as Kitsune [15] and variational autoencoders [16], have effectively addressed the challenge of limited labeled data in network environments. These methods excel in discovering hidden patterns and identifying anomalies without extensive labeled training data. The introduction of Generative Adversarial Networks (GANs) by Ring et al. [17] and their subsequent enhancement by Li et al. [18] has significantly improved model resilience and addressed data imbalance issues in anomaly detection.

Recent advances have seen the emergence of more sophisticated architectures. Graph Neural Networks (GNNs) have been successfully applied to topology-aware security analysis [19,20], while Transformer-based models have demonstrated exceptional capability in capturing long-range dependencies in network traffic [21]. These developments have been complemented by advances in ensemble learning, reinforcement learning, and federated learning approaches [22–24], which collectively enhance model generalization, adaptation, and privacy preservation.

Despite these significant advancements, several fundamental challenges persist in the field of network anomaly detection. The efficient processing and analysis of massive, high-dimensional network traffic data remains a significant technical hurdle [25]. Meeting real-time detection requirements in dynamic environments continues to pose substantial challenges [26], while the need to adapt to evolving attack patterns and unknown threats demands ongoing innovation in detection methodologies [27]. These challenges underscore the importance of developing more robust and adaptive detection frameworks that can effectively operate in complex, evolving network environments.

The landscape of network security detection continues to evolve with significant advancements in deep learning approaches. Recent works have demonstrated promising results in using various neural architectures for anomaly detection. Wang et al. [28] proposed a multi-strategy improved model for environmental monitoring that highlighted the importance of adaptive techniques in dynamic environments, while their work on wireless sensor deployment optimization [29] established the significance of balanced resource allocation in network systems. More directly relevant to our domain, Wang et al. [30] developed a CNN-BiLSTM approach for industrial intrusion detection, achieving notable improvements in accuracy over traditional methods. However, several critical challenges remain unaddressed in the current literature: (1) limited integration of spatial and temporal features, (2) inadequate adaptation to concept drift, and (3) imbalance between detection accuracy and computational efficiency.

This investigation proposes an innovative, synergistic deep learning framework to address these contemporary challenges in anomaly detection. The framework integrates the salient features of CNNs, LSTM and Transformer models, augmented by self-learning mechanisms. This amalgamation aims to enhance the model’s generalization capabilities and operational efficiency, thereby advancing the state-of-the-art in identifying complex, evolving network anomalies. Specifically, the main objectives of this study are as follows,

1. The proposed integrated neural architecture synergistically captures and analyzes both spatial configurations and temporal evolution of network traffic patterns, significantly enhancing the model’s capacity for comprehensive anomaly detection;
2. The adaptive learning mechanisms developed enable the model to continuously respond to emerging and evolving cyber threats, demonstrating improved resilience against novel attack vectors;
3. The optimization strategies implemented for computational efficiency and model scalability, coupled with preservation of anomaly detection accuracy, ensure robust performance across diverse network landscapes and data scales, meeting real-time detection requirements in large-scale environments.

Through experimental verification, on the UNSW-NB15 [31] and CICIDS2017 [32] benchmark dataset, the following main conclusions are drawn:

1. The proposed CNN-LSTM and LSTM-Transf-ormer hybrid model is superior to existing methods in detection accuracy and efficiency, and effectively integrates spatial and temporal feature analysis;
2. The self-learning mechanism introduced significantly improves the detection ability of the model to new and unknown attacks, and enhances the adaptability of the system;
3. Compared with traditional methods, our method shows better generalization ability and computational efficiency, and can meet the real-time detection needs in large-scale network environment.

These findings not only advance the technical capabilities in network traffic analysis but also provide new directions for developing more intelligent and adaptive network security defense systems. The remainder of this paper is organized as follows: the Preliminaries section presents the relevant background and materials. The Proposed framework section details the proposed methodology. The Experiments section describes the experimental setup and results. Finally, the Conclusions section concludes the paper with discussions on future research directions.

## Preliminaries

### Deep learning architectures for network traffic analysis

Modern deep learning architectures have revolutionized network traffic analysis through their ability to automatically extract complex patterns from high-dimensional data [33]. The evolution of these architectures has enabled increasingly sophisticated approaches to anomaly detection [34], particularly in handling the complex, multi-dimensional nature of network traffic data [35].

Convolutional Neural Networks (CNNs) have demonstrated remarkable capabilities in capturing spatial correlations within network traffic features. For an input traffic sequence $X\in {\mathbb{R}}^{T\times F}$ representing T timesteps and F features, CNN applies hierarchical feature extraction through convolution operations: X'=Conv(X)=σ(W*X+b), where W represents learnable filters and *σ* is a nonlinear activation function. This architecture’s inherent ability to learn hierarchical features has proven particularly effective in identifying spatial patterns characteristic of network attacks [36], eliminating the need for manual feature engineering that often limits traditional approaches.

The temporal aspects of network traffic necessitate architectures capable of capturing sequential dependencies [37]. Long Short-Term Memory (LSTM) networks address this challenge through a sophisticated gating mechanism that effectively prevents gradient vanishing in processing long sequential data [38]. The core operations of LSTM are governed by (1):

$$i_{t}=\sigma \left(W_{i}\cdot \left[h_{t-1},x_{t}\right]+b_{i}\right)$$ (1)

$$f_{t}=\sigma \left(W_{f}\cdot \left[h_{t-1},x_{t}\right]+b_{f}\right)$$ (2)

$$o_{t}=\sigma \left(W_{o}\cdot \left[h_{t-1},x_{t}\right]+b_{o}\right)$$ (3)

$${\tilde{C}}_{t}=\tanh\left(W_{C}\cdot \left[h_{t-1},x_{t}\right]+b_{C}\right)$$ (4)

$$C_{t}=f_{t}\times C_{t-1}+i_{t}\times {\tilde{C}}_{t}$$ (5)

In this formulation, the gating mechanisms ($i_{t},f_{t},o_{t}$) control information flow through the network, enabling the model to capture long-term dependencies crucial for detecting evolving attack patterns [39]. The LSTM’s ability to maintain and update internal state makes it particularly effective in identifying attacks that manifest over extended time periods [40].

The Transformer architecture represents a significant advancement in sequence modeling through its introduction of self-attention mechanisms. The core attention operation is defined as (6)

$$\text{Attention}(Q,K,V)=\text{softmax}(\frac{QK^{T}}{\sqrt{d_{k}}})V$$ (6)

where Q,K and V represent learnable transformations of the input features. This mechanism enables the model to dynamically capture global dependencies across entire traffic sequences, offering unprecedented capabilities in identifying complex attack patterns that manifest across different time scales [41]. The multi-head attention mechanism further enhances this capability by allowing the model to attend to different aspects of the input simultaneously [42].

### Data drift detection in network security

Network traffic patterns exhibit inherent temporal evolution, manifesting as data drift when the distribution of traffic patterns shifts between the training (source) domain *p*_*s*_(*x*) and the operational (target) domain *p*_*t*_(*x*). This phenomenon poses a fundamental challenge to the long-term effectiveness of anomaly detection systems [43]. The performance degradation under drift can be quantified through the accuracy metric (7):

$$\text{Acc}(f)=𝔼(x,y)∼p_{t}[1f(x)=y]$$ (7)

which typically decreases when $p_{s}\neq p_{t}$.

The detection and adaptation to data drift have emerged as critical components of robust network security systems. However, traditional single-metric drift detection methods face a fundamental limitation in network security contexts: the temporal decoupling between statistical significance and functional impact. Methods like ADWIN [44] may trigger unnecessary adaptations for benign traffic changes, while performance-only approaches like DDM [45] may miss early indicators of sophisticated attacks. Our multi-metric approach addresses this challenge by requiring simultaneous validation across statistical, performance, and distributional dimensions, enabling systems to maintain detection efficacy through adaptive learning while preserving knowledge of previously encountered attack signatures.

### Self-learning mechanisms

Self-learning mechanisms enable anomaly detection systems to adapt autonomously using unlabeled traffic data, addressing the practical constraints of obtaining labeled samples in operational environments. The autoencoder paradigm exemplifies this approach, learning normal traffic patterns through the optimization objective (8):

$$\min\theta ,\phi 𝔼x∼p(x)[|x-g_{\phi }(f\theta (x)){|}^{2}]$$ (8)

where $f_{\theta }$ and $g_{\phi }$ represent encoder and decoder functions respectively [46]. This framework enables the identification of anomalies through reconstruction error analysis, providing a foundation for unsupervised adaptation to evolving traffic patterns.

Recent studies have introduced more sophisticated self-learning approaches, including contrastive learning for feature space optimization. The contrastive learning objective can be formulated as (9):

$${ℒ}_{i,j}=-\log\frac{\exp(\text{sim}({𝐳}_{i},{𝐳}_{j})/\tau )}{\sum_{k=1}^{2N}1\left[k\neq i\right]\exp(\text{sim}({𝐳}_{i},{𝐳}_{k})/\tau )}$$ (9)

where $z_{i},z_{j}$ represents two augmented views of the same traffic sample, and $z_{k}$ represents the other negative samples. This approach enables the model to learn discriminative features without explicit labels.

Furthermore, adaptive threshold mechanisms have been developed to dynamically adjust detection boundaries. The adaptive threshold $\theta_{t}$ at time t can be updated according to (10):

$$\theta_{t}=\mu_{t}+\alpha \sigma_{t}$$ (10)

where $\mu_{t}$ and $\sigma_{t}$ represent the running mean and standard deviation of the anomaly scores, and *α* is a sensitivity parameter that controls the detection boundary. The integration of these self-learning mechanisms with deep learning architectures has opened new possibilities for autonomous, adaptive anomaly detection systems. The combination of representation learning and adaptive mechanisms enables continuous model refinement while maintaining robustness to concept drift, a crucial capability in operational network security environments [47].

## Proposed framework

### Framework overview

The rapid evolution of cyber threats presents unprecedented challenges to network security systems, characterized by sophisticated attack patterns and dynamic behavior changes. Traditional approaches often struggle with these challenges, particularly in scenarios involving zero-day attacks and concept drift. We propose a novel hybrid deep learning framework, as illustrated in S1 Fig, that integrates specialized neural architectures with adaptive learning mechanisms to address these challenges effectively.

Our framework employs a hierarchical detection paradigm that decomposes network anomaly detection into two specialized stages, each optimized for specific aspects of the detection task, with detailed architecture specifications shown in Table 1. The first stage utilizes a CNN-LSTM architecture for binary classification, leveraging CNN’s capability to extract spatial correlations while LSTM captures temporal dependencies. The second stage employs an LSTM-Transformer architecture for fine-grained attack classification, where the Transformer’s self-attention mechanism enables complex pattern recognition across different traffic characteristics.

**Table 1:** Model architecture specifications for two-stage deep learning framework.

First Stage: CNN-LSTM		Second Stage: LSTM-Transformer	
Layer	Parameters	Layer	Parameters
Conv1D	filters = 64, kernel = 3	BiLSTM	units = 256
LSTM	units = 256	Transformer	heads = 8, layers = 2
Output Shape	T × 64	Output Shape	T × 512

Table notes: T represents the number of timesteps in the input sequence. The output shapes indicate the dimensionality of feature representations at each stage.

### Two-stage deep model fusion

The CNN-LSTM module serves as the framework’s initial detection layer, transforming network traffic features through hierarchical convolution operations. The CNN component maps input data into a structured feature space where spatial correlations between traffic attributes are captured. For a traffic sequence $X\in {\mathbb{R}}^{T\times F}$ with T timesteps and F features, the CNN applies feature extraction through convolution operations, enabling the identification of local patterns indicative of anomalous behavior.

The LSTM-Transformer module builds upon the binary classification results, processing identified anomalous traffic for detailed attack type classification. This stage employs bidirectional LSTM layers to create rich temporal representations, followed by a Transformer encoder that uses multi-head self-attention to capture global dependencies. This architecture enables the model to identify subtle differences between attack types while maintaining computational efficiency.

This hierarchical integration addresses the multi-scale nature of network traffic anomalies that single-model approaches cannot handle effectively. Network attacks manifest across different temporal and spatial scales—from microsecond-level packet timing anomalies to hour-long distributed attack campaigns. Independent CNN processing captures local traffic correlations but misses extended attack sequences, while standalone LSTM analysis models temporal patterns but struggles with high-dimensional feature interactions. Direct Transformer application to raw network traffic creates computational overhead that prevents real-time processing in operational environments.

The two-stage architecture optimizes detection efficiency by matching computational complexity to analysis requirements. Binary anomaly detection requires less sophisticated pattern analysis than fine-grained attack classification, making CNN-LSTM processing suitable for initial filtering. The subsequent LSTM-Transformer stage applies intensive analysis only to suspected anomalous traffic, achieving comprehensive detection while maintaining operational feasibility. This design enables the framework to process typical network speeds of 10-500 Mbps while preserving detection accuracy for complex attack patterns.

### Adaptive learning mechanism

The framework implements its self-learning capability through an adaptive learning mechanism that enables autonomous response to evolving attack patterns while preserving knowledge of known threats. As illustrated in S2 Fig, this mechanism operates through multi-metric drift detection and selective model updating. The system monitors potential distribution shifts between the source domain *p*_*s*_(*x*) and target domain *p*_*t*_(*x*) through statistical testing and performance monitoring, triggering adaptation when significant changes are detected. This adaptive learning mechanism represents the core of our framework’s self-learning ability, allowing it to continuously improve detection accuracy without human intervention as new attack patterns emerge.

Our multi-metric drift detection method employs a comprehensive approach to identify distribution shifts in network traffic patterns. The drift detection is formulated as follows:

Given a reference distribution Dref from the source domain *p*_*s*_(*x*) and current traffic distribution D from the potential target domain *p*_*t*_(*x*), we compute a composite drift score using three complementary metrics:

1. Statistical divergence (Dstat): We employ the Kolmogorov-Smirnov test to measure the statistical significance of distribution differences between reference and current traffic features (11):
  Dstat=KS_test(Dref,D) (11)
2. Performance degradation (Dperf): This metric quantifies the decline in model performance when applied to new traffic patterns (12):
  $$\text{Dref}=1-\frac{\text{P}erformance(M,D)}{Performance(M,Dref)}$$ (12)
  where Performance() represents the model’s F1-score on the respective datasets.
3. Distribution shift (Ddist): We calculate the Jensen-Shannon divergence between feature distributions to capture subtle shifts that might not be reflected in immediate performance drops (13):
  $$Ddist=JSD\left(Dref||D\right)=\frac{1}{2}\times \left[KL\left(Dref||M\right)+KL\left(D||M\right)\right]$$ (13)
  where $M=\frac{1}{2}\times \left(Dref+D\right)$ and KL represents the Kullback-Leibler divergence.

These three metrics capture different aspects of potential drift: Dstat reflects statistically significant changes, Dperf indicates functional impact on model performance, and Ddist measures intrinsic distribution changes. The composite drift score is calculated as a weighted combination (14):

drift_score=α·Dstat+β·Dperf+γ·Ddist (14)

where *α*, *β*, and *γ* are importance weights determined through experimental validation. A drift is detected when drift_score exceeds threshold τd, triggering the model update process. This multi-metric approach provides robustness against false positives while ensuring sensitivity to meaningful distribution shifts across diverse network environments.

Model updates employ Elastic Weight Consolidation to preserve important parameters while adapting to new patterns. When a concept drift is detected, our framework initiates a systematic recovery process through the following steps:

1. Important parameter identification: The framework computes the Fisher Information Matrix F to identify parameters crucial for maintaining existing knowledge (15):
  $$F_{i}=E\left[{\left(\frac{\partial logp\left(y|x,\theta \right)}{\partial \theta_{i}}\right)}^{2}\right]$$ (15)
  where $\partial \theta_{i}$ represents individual model parameters and p(y|x,θ) is the probability of predicting class y given input x.
2. Constrained optimization: The model parameters are updated with a loss function that balances new learning objectives against the preservation of existing knowledge (16):
  $$L\left(\theta \right)=Lnew\left(D,\theta \right)+\lambda \sum F_{\text{i}}{\left(\theta_{\text{i}}-\theta_{\text{i}}*\right)}^{2}$$ (16)
  where $\theta_{\text{i}}*$ represents the original parameter values before update, and *λ* controls the strength of knowledge preservation.

This approach enables continuous adaptation to emerging threats while maintaining detection capability for known attack patterns. Through extensive experimentation, we determined that this process enables the model to recover baseline performance within 23.4 ± 0.20 hours post-drift detection, significantly faster than traditional retraining approaches.

The integration of these components creates a robust framework capable of maintaining high detection accuracy across diverse network environments while efficiently processing high-dimensional traffic data. The framework’s effectiveness is thoroughly evaluated through comprehensive experiments detailed in Sect 4.

### Framework implementation

Our framework processes network traffic data through a sophisticated pipeline that transforms raw packet data into comprehensive traffic representations. The core components of our framework and their primary functions are summarized in Table 2.

**Table 2:** Core components and their functions.

Component	Primary Function	Key Features
Feature Processor	Traffic Analysis	Flow aggregation, Protocol parsing
CNN-LSTM	Binary Detection	Spatial-temporal pattern recognition
LSTM-Transformer	Attack Classification	Context-aware pattern analysis
Self-Learning Module	Adaptation	Drift detection, Knowledge preservation

The feature processing begins with traffic flow aggregation, where individual packets are grouped into meaningful flows based on their temporal and spatial relationships. This aggregation process considers both protocol-specific characteristics and statistical properties of the traffic patterns, enabling the framework to capture complex attack signatures that manifest across multiple packets or flows.

The CNN-LSTM module employs a hierarchical feature extraction approach, where convolutional layers progressively identify increasingly abstract patterns in the traffic data. Through careful architectural design, the CNN component captures local feature correlations while maintaining computational efficiency. The LSTM layers then analyze these features in their temporal context, enabling the detection of sophisticated attacks that evolve over time. The integration of these components allows the model to maintain high detection accuracy while processing traffic data in real-time.

The LSTM-Transformer module builds upon this foundation through its sophisticated attention mechanisms. By incorporating bidirectional LSTM encoding with multi-head self-attention, the module can identify subtle relationships between different aspects of anomalous traffic. This capability is particularly crucial for distinguishing between various attack types that may share similar surface characteristics but differ in their underlying patterns or intentions.

A critical aspect of our implementation is the self-learning mechanism’s integration with both detection stages. This mechanism continuously monitors the traffic distribution and model performance, automatically detecting when the current detection patterns need to be updated. The update process employs Elastic Weight Consolidation to prevent catastrophic forgetting, ensuring that the model maintains its ability to detect known attack patterns while adapting to new threats.

The framework’s effectiveness stems from the synergistic interaction between these components, enabling robust performance across diverse network environments and attack scenarios. Algorithm 1 illustrates the core detection process, while Algorithm 2 and Algorithm 3 detail the adaptive learning and drift detection mechanisms respectively. These algorithms collectively enable the framework to maintain high detection accuracy while efficiently processing high-dimensional traffic data in real-time environments.

**Algorithm 1. Two-stage network anomaly detection.**

Input: $\text{Network traffic sequence }X=\{x_{1},x_{2},...,x_{t}\}$

Output: Traffic classification result (normal/attack)

1: Initialize model parameters $\theta_{CNN}$, $\theta_{LSTM}$, $\theta_{Trans}$

3: for each traffic window *w* in *X* do

4:   $F=CNN\_extract(w,\theta_{CNN})$

5:   $T=LSTM\_analyze(F,\theta_{LSTM})$

6:   score=binary_classify(T)

7:   if *score* > *threshold* then

9:    S=BiLSTM_encode(w)

10:    $A=Transformer\_analyze(S,\theta_{Trans})$

11:    return attack_classify(A)

12:   end if

13: end for

14: return “normal”

**Algorithm 2. Adaptive learning mechanism.**

Input: Current model M, New data batch D

Output: $\text{Updated model }M^{\prime }$

1: $D_{stat}=KS\_test(D_{ref},D)$

2: $D_{perf}=performance\_drop(M,D)$

3: $D_{dist}=distribution\_shift(D_{ref},D)$

4: $drift\_score=\alpha \cdot D_{stat}+\beta \cdot D_{perf}+\gamma \cdot D_{dist}$

5: if $drift\_score>\tau_{d}$ then

6:   F=compute_fisher_matrix(M)

7:   $\theta^{*}=get\_important\_params(M,F)$

8:   $L(\theta )=L_{new}(D,\theta )+\lambda \sum F_{i}(\theta_{i}-\theta_{i}^{*}{)}^{2}$

9:   $M^{\prime }=optimize(L(\theta ))$

10:    $validate(M^{\prime })$

11:    return $M^{\prime }$

12: end if

13: return *M*

**Algorithm 3. Real-time pattern analysis.**

Input: Traffic sequence X, Window size w, Stride s

Output: Pattern detection result R

1: Initialize pattern buffer B

2: for each window *W* in slide(X,w,s) do

3:   F=extract_features(W)

4:   P=analyze_patterns(F)

5:   update_buffer(B,P)

6:   if detect_anomaly(B) then

7:    trigger_analysis(B)

8:   end if

9: end for

10: return aggregate_results(B)

## Experiments

### Experimental setup

Our experimental evaluation employs two widely-used network security datasets that represent different network environments and attack scenarios. The UNSW-NB15 dataset encompasses over 2.5 million records with 49 features, featuring nine distinct attack types generated in a controlled environment. The CICIDS2017 dataset contains approximately 2.8 million network flows with 78 features, captured from real-world network traffic and including 14 contemporary attack patterns. A detailed comparison of these datasets is presented in Table 3.

**Table 3:** Dataset characteristics comparison.

Characteristic	UNSW-NB15	CICIDS2017
Total Records	2,540,044	2,830,743
Attack Types	9	14
Normal Traffic	68.1%	83.3%
Feature Count	49	78
Collection Period	31 hours	5 days

The data preprocessing pipeline consists of several systematic stages designed to enhance data quality and model performance:

1. Data cleaning: We addressed missing values using attribute-specific strategies: categorical features were filled with the most frequent value, while numerical features were imputed using the median value to minimize the influence of outliers. Duplicate records were removed to prevent biasing the model toward redundant patterns.
2. Normalization: Numerical features were standardized using z-score normalization (*μ* = 0, *σ* = 1) to ensure balanced contribution of features with different scales (17):
  $$X\_norm=\frac{X-\mu }{\sigma }$$ (17)
  This step is particularly important for the gradient-based optimization in our deep learning models.
3. Outlier handling: We employed the Interquartile Range (IQR) method (*τ* = 1.5) to detect and address outliers (18):
  Lower bound=Q1−τ×IQR (18)
  Upper bound=Q3+τ×IQR (19)
  Values outside these bounds were capped rather than removed, preserving data points while limiting their potential to skew the model training.
4. Feature selection and dimensionality reduction: We implemented a multi-stage feature selection process:
  - Initial filtering through correlation analysis (threshold = 0.1) to eliminate highly redundant features;
  - Random Forest importance ranking to identify the most discriminative features;
  - Sequential Forward Selection with cross-validation to optimize the feature subset while minimizing information loss.

Through comprehensive feature analysis, we identified the most significant contributors to attack detection performance across both datasets, as shown in S3 Fig. Flow duration and packet size consistently emerge as the most crucial features, with importance scores above 0.80 across both datasets, while protocol-specific characteristics show varying degrees of importance depending on the network environment.

To address potential redundancy in the feature space, we employed Principal Component Analysis (PCA) for the LSTM-Transformer stage, retaining components that explained 95% of the variance while reducing computational complexity. For the CNN-LSTM stage, we preserved the original feature space to maintain interpretability and spatial correlations.

This systematic feature engineering approach resulted in optimized feature sets of 35 and 45 features for UNSW-NB15 and CICIDS2017 respectively, balancing model performance with computational efficiency. The selected features encompass temporal characteristics, volumetric properties, and protocol-specific attributes, providing a comprehensive representation of network traffic patterns.

All experiments were conducted using TensorFlow 2.15.0 on a system equipped with Google Colaboratory environment, ensuring consistent evaluation conditions across all comparative analyses.

### Performance evaluation

Our framework demonstrates superior performance across both datasets in binary anomaly detection and multi-class attack classification tasks. On the UNSW-NB15 dataset, the framework achieves an F1-score of 0.9778 for binary detection and 0.9632 accuracy for attack classification, representing significant improvements over existing approaches. The framework’s effectiveness is further validated on the CICIDS2017 dataset, achieving F1-scores of 0.9695 and 0.9528 for binary and multi-class tasks respectively. A comprehensive comparison with existing methods is presented in Tables 4 and 5.

**Table 4:** Comparative performance analysis on UNSW-NB15 datasets.

Method	UNSW-NB15			
	Binary (F1)	Binary (ACC)	Multi-class (F1)	Multi-class (ACC)
Our Framework	**0.9778**	**0.9632**	**0.9632**	**0.9528**
CNN-LSTM [48]	0.9716	0.9443	0.9390	0.8290
TGAN-AD [49]	–	0.9590	0.9050	–
CTGAN [50]	0.9518	0.9800	0.9390	0.9500
Transformer-only	0.9690	0.9410	0.9550	0.9320
Traditional ML	0.9256	0.9156	0.9189	0.9023

**Table 5:** Comparative performance analysis on CICIDS2017 datasets.

Method	CICIDS2017	
	Binary (F1)	Multi-class (F1)
Our Framework	**0.9695**	**0.9528**
CNN-LSTM [48]	0.9585	0.9312
TGAN-AD [49]	-	0.9125
CTGAN [50]	0.9486	0.9284
Transformer-only	-	-
Traditional ML	0.9156	0.9023

The results demonstrate that our integrated CNN-LSTM-Transformer framework outperforms both single-paradigm models and alternative hybrid architectures. Notably, while CTGAN shows competitive performance in binary detection, it underperforms in the more challenging multi-class scenario compared to our approach. The pure Transformer model, despite its theoretical advantages in capturing long-range dependencies, achieves lower F1-scores than our hybrid approach in both binary and multi-class detection tasks. This suggests that the integration of convolutional operations for spatial feature extraction with sequential and attention-based models for temporal analysis provides complementary capabilities that pure attention-based approaches cannot fully replicate.

The framework’s adaptability is further illustrated in S4 Fig, which shows the accuracy comparison between adaptive and non-adaptive models over a 15-day period. The adaptive model (blue line) maintains consistently high accuracy around 0.95 despite concept drift events (marked by red vertical lines), while the non-adaptive model (green line) shows significant performance degradation after each drift occurrence. This demonstrates the effectiveness of our self-learning mechanism in preserving model performance under evolving network conditions.

The superior performance can be attributed to several key factors:

1. The two-stage detection architecture effectively captures both general anomaly patterns and specific attack characteristics;
2. The integration of CNN-LSTM and LSTM-Transformer modules provides comprehensive feature analysis at multiple scales;
3. The self-learning mechanism enables rapid adaptation to emerging threats while maintaining detection accuracy.

Particularly noteworthy is the framework’s consistent performance across both datasets, demonstrating its robustness and generalization capability. The performance improvement is more pronounced in complex attack scenarios and zero-day attack detection, where the framework achieves a 92.8% detection rate for previously unseen attack patterns.

### Model analysis

To comprehensively evaluate our framework’s design choices and component contributions, we conducted extensive ablation studies on both datasets. The ablation results, presented in Tables 6 and 7, demonstrate the significant contribution of each component to the framework’s overall performance.

**Table 6:** Ablation study results on UNSW-NB15 dataset.

Model Variant	Binary F1	Multi-class F1	Recovery Time (h)
Base(CNN-LSTM)	0.9412	–	–
+ Transformer	0.9544	0.9428	–
+ Self-learning	**0.9778**	**0.9632**	23.4 ± 0.20

**Table 7:** Ablation study results on CICIDS2017 dataset.

Model Variant	Binary F1	Multi-class F1	Recovery Time (h)
Base(CNN-LSTM)	0.9385	–	–
+ Transformer	0.9455	0.9312	–
+ Self-learning	**0.9695**	**0.9528**	24.1 ± 0.25

The ablation results illuminate the detection challenges that each component addresses and reveal why their combination proves necessary for comprehensive network anomaly detection. The base CNN-LSTM model achieves F1-scores of 0.9412 and 0.9385, establishing strong detection capability that encounters specific limitations when processing network traffic with distributed attack signatures.

Investigation of misclassified samples reveals that the base model struggles with attacks whose indicators appear across non-contiguous time windows. Consider an advanced persistent threat that establishes legitimate-appearing connections early in a session, maintains dormancy for hours, then exploits these connections for data exfiltration. The CNN-LSTM architecture processes traffic in sequential windows, making correlation of these temporally distant but functionally related events challenging.

The Transformer addition addresses this limitation directly, yielding improvements of 1.32% and 0.70% on the respective datasets. This gain magnitude reflects the Transformer’s role in handling the subset of attacks requiring global temporal correlation rather than replacing the CNN-LSTM’s local processing capabilities. Analysis of newly detected attacks shows that 89% involve multi-stage intrusions where attack indicators span extended time periods with normal traffic interspersed.

The self-learning mechanism produces the most substantial improvements—2.34% and 2.40%—by addressing the pervasive challenge of evolving attack patterns. Without adaptation, model performance degrades by an average of 8.7% over the evaluation period as attack tools and techniques evolve. The self-learning component not only prevents this degradation but enables continuous improvement through exposure to emerging threat patterns.

The integrated framework demonstrates how component interactions create detection capabilities that exceed simple additive combinations. Sequential processing creates synergy where CNN-LSTM output provides structured representations that facilitate Transformer attention computation. Raw traffic features would require the Transformer to simultaneously learn feature extraction and global correlation, reducing both efficiency and accuracy. The CNN-LSTM preprocessing enables the Transformer to focus on pattern correlation across temporal distances.

The self-learning mechanism enables dynamic component weighting based on current traffic characteristics. During periods dominated by local attack patterns, the system emphasizes CNN-LSTM contributions. When multi-stage attacks increase in frequency, Transformer influence grows proportionally. This adaptive weighting prevents over-reliance on any single component while maximizing the strengths of each.

Error recovery operates through the complementary nature of component limitations. CNN-LSTM excels at local pattern recognition but may miss distributed attack signatures. The Transformer captures global correlations but requires structured input to operate efficiently. Self-learning adapts both components to evolving threats but needs stable base detection to identify meaningful patterns. Each component compensates for the others’ constraints while amplifying their capabilities.

Different attack types reveal the specialized value of each component. Volume-based attacks like DDoS exhibit strong local signatures that CNN-LSTM detects with 96.2% accuracy. The Transformer contributes minimal improvement for these attacks since their indicators cluster temporally. Self-learning provides enhancement by adapting to evolving attack tools and amplification techniques. Stealth attacks present the opposite pattern, where advanced persistent threats achieve only 78.4% detection with CNN-LSTM alone, as these attacks deliberately distribute their signatures across time. The Transformer contributes substantial improvement by correlating these distributed indicators, while self-learning recognizes new stealth techniques as they emerge.

Zero-day attacks demonstrate the most variable performance, with CNN-LSTM achieving detection rates between 45-89% depending on similarity to known attack patterns. The Transformer provides moderate assistance through pattern generalization, but self-learning delivers the most significant improvement by rapidly incorporating new attack signatures into the detection model. This analysis demonstrates why each component addresses specific aspects of the network anomaly detection challenge and why their integration creates detection capabilities that no single component can achieve alone.

### Discussion

The experimental results demonstrate both the capabilities and limitations of our proposed framework across different network environments and attack scenarios. Our evaluation on the UNSW-NB15 and CICIDS2017 datasets reveals significant improvements in detection accuracy, with F1-scores of 0.9778 and 0.9695 respectively, representing substantial advances over existing approaches. The framework’s ability to maintain such high performance levels across different network environments suggests robust generalization capabilities, particularly in handling complex attack patterns and previously unseen threats.

The 92.8% zero-day detection rate emerges from the framework’s multi-layered generalization capability and adaptive learning architecture. This performance was evaluated using unseen attack patterns that were temporally separated from training data, representing genuine zero-day scenarios where attack signatures are unavailable during model development.

The detection mechanism operates through three complementary pathways that address different aspects of zero-day threat identification. The CNN-LSTM foundation provides pattern generalization by learning abstract traffic representations that capture attack behaviors independent of specific implementation details. During training, the CNN layers learn to identify spatial correlations in traffic features that indicate malicious intent—such as anomalous packet size distributions, unusual protocol combinations, or irregular timing patterns. These learned representations enable detection of new attacks that exploit similar network vulnerabilities through different technical approaches.

The LSTM component contributes temporal generalization by modeling attack progression patterns that persist across different attack variants. Zero-day attacks often follow recognizable phases—reconnaissance, exploitation, and data exfiltration—even when specific techniques differ from known attacks. The LSTM’s memory mechanism captures these temporal signatures, enabling identification of attack sequences that follow familiar strategic patterns despite employing novel tactical approaches. The Transformer component provides the most sophisticated zero-day detection capability through its attention-based correlation of distant traffic events. Many advanced zero-day attacks employ distributed strategies where attack indicators appear across non-contiguous time periods to evade detection. The Transformer’s self-attention mechanism enables correlation of these distributed signatures, identifying attack patterns that span extended temporal ranges. This capability proves particularly valuable for detecting advanced persistent threats that establish legitimate-appearing connections before exploiting them for malicious purposes.

The self-learning mechanism enhances zero-day detection through continuous model refinement based on operational feedback. When the system encounters borderline cases where initial classification confidence is low, the multi-metric drift detection evaluates whether these cases represent genuine anomalies requiring investigation. Confirmed zero-day attacks trigger focused parameter updates that incorporate new attack signatures while preserving existing knowledge through Elastic Weight Consolidation. This creates an evolutionary learning process where the system’s zero-day detection capability improves through exposure to diverse threat patterns.

Compared to traditional approaches, our framework addresses fundamental limitations in zero-day detection methodologies. Signature-based systems fail completely against zero-day attacks due to their reliance on predefined attack patterns. Single-model anomaly detection systems, while capable of identifying novel patterns, typically generate excessive false positives that make operational deployment impractical. The CNN-LSTM baseline in our ablation study achieves 0.9412 F1-score, representing strong detection capability that nonetheless misses complex attacks requiring global temporal correlation.

Pure deep learning approaches without adaptive capabilities suffer from concept drift, where performance degrades as attack patterns evolve beyond the training distribution. Our self-learning mechanism directly addresses this limitation, enabling sustained performance against evolving zero-day threats. The 23.4-hour recovery time following drift detection demonstrates the system’s ability to rapidly incorporate new threat intelligence while maintaining operational effectiveness.

The framework’s zero-day detection capability also demonstrates robustness against adversarial evasion attempts. The multi-component architecture creates multiple detection pathways that attackers must simultaneously evade to avoid detection. An attack that successfully evades CNN-based spatial analysis may still be detected through LSTM temporal analysis or Transformer global correlation. This architectural redundancy, combined with continuous learning adaptation, creates a moving target that complicates adversarial attack development.

The two-stage detection architecture balances detection accuracy with computational efficiency through selective processing. Table 8 shows the framework processes 1250 samples per second for binary classification and 950 samples per second for attack classification, with memory usage of 2.8 GB and 3.5 GB respectively.

**Table 8:** Computational efficiency analysis.

Processing Stage	Throughput (samples/s)	Memory Usage (GB)
Binary Detection	1250 ± 45	2.8 ± 0.2
Attack Classification	950 ± 35	3.5 ± 0.3

The LSTM-Transformer component operates with $\text{O}(T^{2}d)$ complexity for self– attention and $\text{O}(Td^{2})$ for LSTM processing, where T represents sequence length and d denotes feature dimensions. This complexity remains manageable because only 8-12% of network traffic typically requires the computationally intensive second-stage analysis. The CNN-LSTM stage filters traffic at line speed, reserving Transformer processing for suspected anomalous patterns.

The framework’s processing capacity suits typical network monitoring requirements. The two-stage design enables efficient resource utilization by applying intensive Transformer processing only to traffic requiring detailed analysis. High-throughput environments can maintain real-time performance through batch processing and GPU acceleration for the LSTM-Transformer component when needed.

Memory utilization scales linearly with batch size rather than sequence length, as both LSTM hidden states and attention weights operate within fixed window sizes. The framework maintains detection performance above 95% across different batch sizes, enabling flexible deployment based on available computational resources. Optimization techniques including attention pruning and weight quantization can reduce computational requirements by 20-30% and memory usage by 50% respectively, with minimal impact on detection accuracy.

## Practical implications

This research offers several actionable insights for operational network security implementations. The framework’s practical applicability spans multiple dimensions of cybersecurity operations:

The adaptive recovery mechanism provides a defined resilience metric for security operations planning, enabling organizations to quantify their vulnerability window and allocate resources accordingly. This represents a significant operational advantage over systems requiring manual reconfiguration after detecting novel attacks.

For zero-day threat mitigation, the framework’s 92.8% detection capability can be implemented through an integration protocol where suspicious traffic identified by the CNN-LSTM component triggers escalated scrutiny and potential containment actions before attack vectors can fully materialize.

Resource provisioning can be precisely calculated using the computational efficiency metrics in Table 8. With 1250 samples/second processing capacity in binary detection mode, organizations can determine hardware requirements as a direct function of their network traffic volume, optimizing capital expenditure while maintaining detection efficacy.

The hierarchical detection architecture enables implementation of tiered security response policies, where routine traffic undergoes baseline screening while anomalous patterns receive comprehensive analysis through the LSTM-Transformer component, creating an efficient resource allocation model aligned with risk-based security management principles.

For organizations with established security operations centers (SOCs), the framework provides multiple integration pathways with existing cybersecurity infrastructure. The CNN-LSTM component serves as an intelligent preprocessing layer for traditional intrusion detection systems, reducing computational overhead while extending detection coverage to unknown threats. Integration with SIEM platforms enables the LSTM-Transformer component to provide enriched threat analysis and automated incident classification. The self-learning mechanism operates through feedback loops where analyst confirmations automatically refine detection parameters, reducing operational overhead while accelerating adaptation to emergent threats.

The modular architecture supports distributed deployment scenarios where organizations can coordinate threat detection across network boundaries through federated learning approaches, enabling collaborative security intelligence while preserving data privacy. Multi-site implementations can deploy specialized framework instances for different network segments, with centralized coordination providing comprehensive threat visibility across diverse infrastructure environments.

These implementation pathways demonstrate the framework’s versatility across diverse operational contexts, providing tangible cybersecurity enhancements for organizations regardless of their security maturity level.

## Conclusions

This research advances the field of network security through a novel approach to anomaly detection, introducing a synergistic framework that combines deep learning architectures with autonomous learning capabilities. Our work demonstrates that integrating spatial-temporal feature analysis with adaptive learning mechanisms can significantly enhance the robustness and reliability of network security systems. The framework’s success across different network environments and attack scenarios validates the effectiveness of our hierarchical detection approach and highlights the potential of self-learning systems in cybersecurity applications. Beyond the immediate technical achievements, this research opens new pathways for developing more intelligent and autonomous security systems. The framework’s ability to maintain effectiveness while adapting to emerging threats represents a significant step toward self-evolving cybersecurity solutions. This advancement is particularly relevant in the context of increasingly sophisticated cyber threats and the growing complexity of network infrastructures. Our findings suggest that future cybersecurity systems will benefit from deeper integration of adaptive learning mechanisms and context-aware detection strategies.

The broader implications of this work extend to the practical aspects of network security management, where the balance between detection accuracy and operational efficiency remains a critical challenge. Our research demonstrates that through careful architectural design and innovative learning mechanisms, it is possible to achieve both high detection accuracy and practical deployment efficiency. This understanding will guide future developments in autonomous security systems, contributing to the evolution of more resilient and adaptable cybersecurity solutions.

Despite these contributions, we recognize several limitations that indicate promising research directions. Further validation in specialized network environments such as industrial control systems and IoT deployments would enhance generalizability. Reducing dependency on labeled training data through transfer learning and few-shot learning techniques represents another critical area for investigation. Additionally, enhancing the framework’s resilience against adversarial evasion attempts and improving model explainability would increase practical utility for security analysts. These areas of future research align with our vision of increasingly autonomous, efficient, and trustworthy anomaly detection systems capable of meeting emerging cybersecurity challenges.

## Supporting information

S1 Fig The general framework. (TIF)

S2 Fig Visualization of the adaptive learning process showing drift detection and model updating. (TIF)

S3 Fig Feature importance distribution. (TIF)

S4 Fig A line graph showing the accuracy performance of adaptive and non-adaptive models about the 15-day period. (TIF)
