# Supplementary figures and images for: Self-learning model fusion for network anomaly detection: A hybrid CNN-LSTM-transformer framework
Source: PLoS One. 2025 Oct 29;20(10):e0332502. doi: 10.1371/journal.pone.0332502 (PMC12571298; doi:10.1371/journal.pone.0332502)

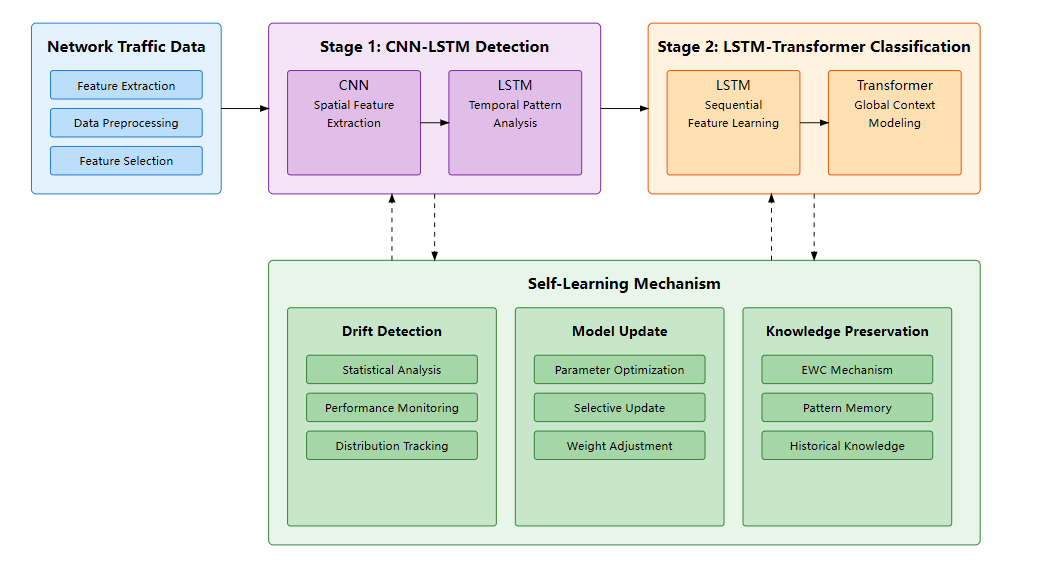

Supplement: S1 Fig — (TIF) [file pone.0332502.s001.tif]

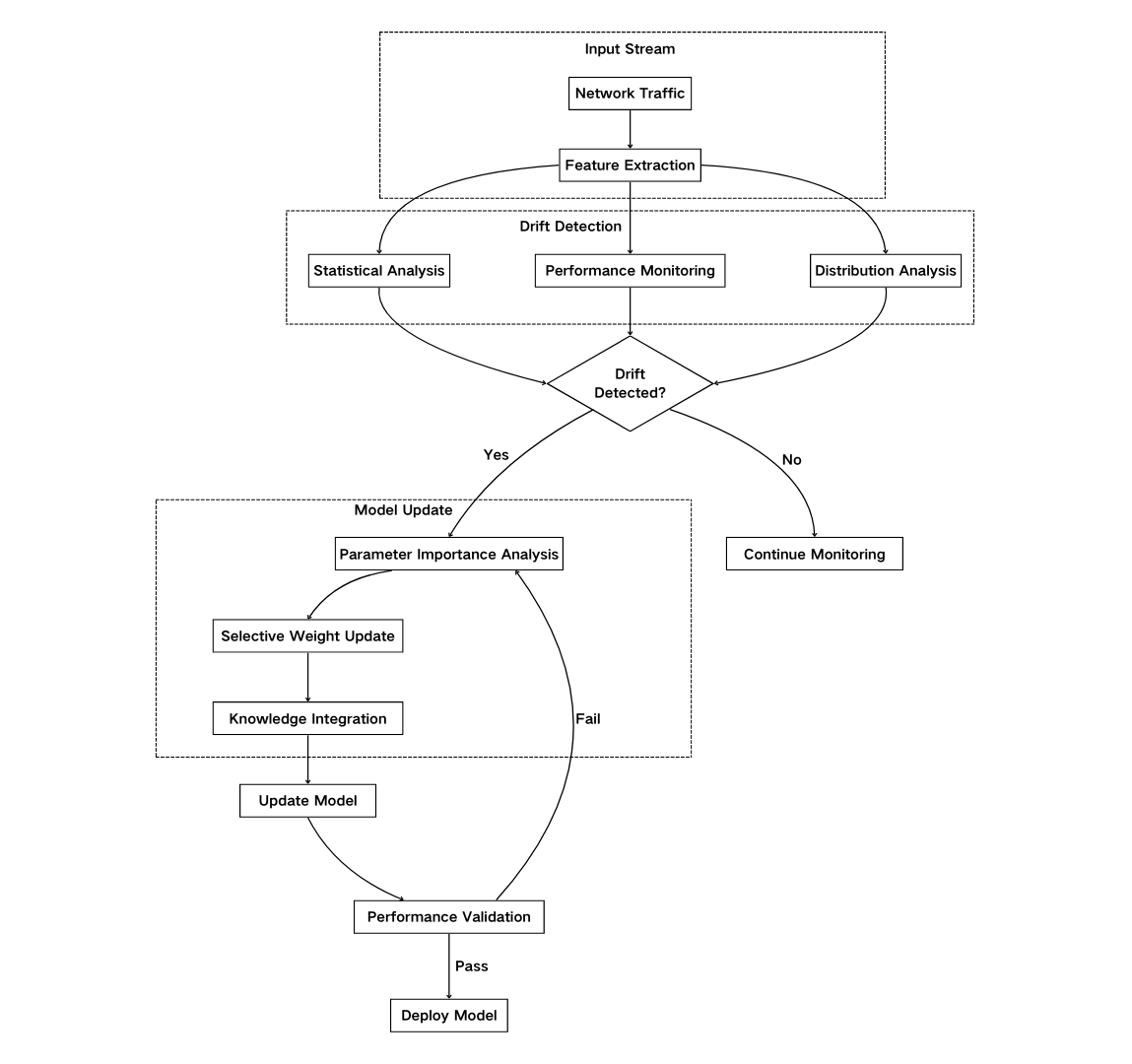

Supplement: S2 Fig — (TIF) [file pone.0332502.s002.tif]

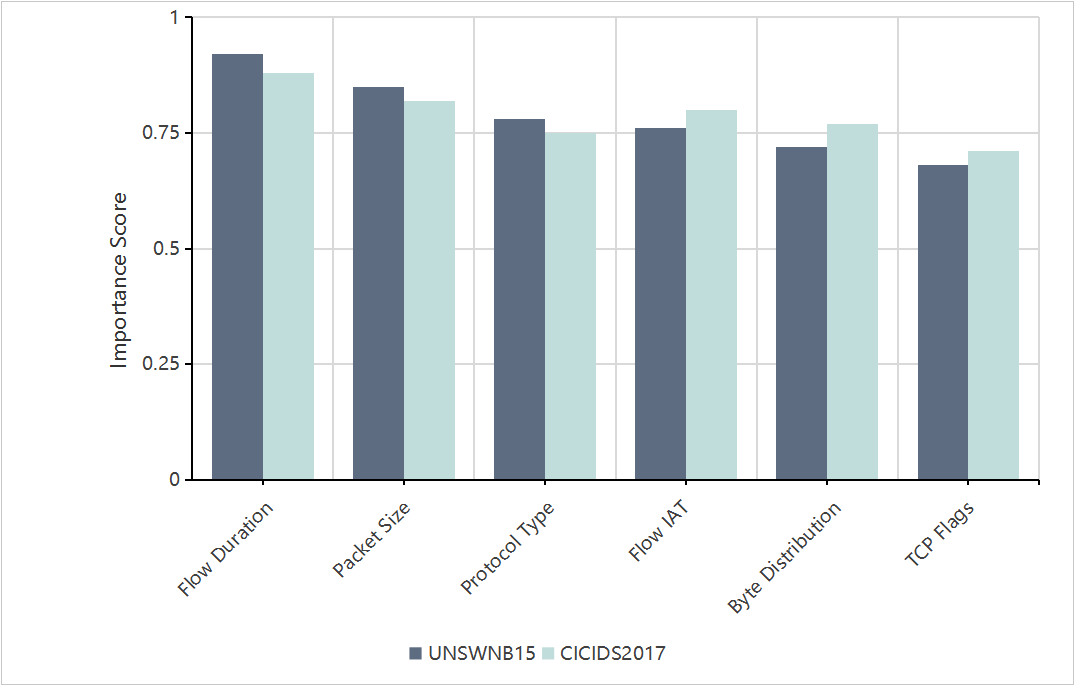

Supplement: S3 Fig — (TIF) [file pone.0332502.s003.tif]

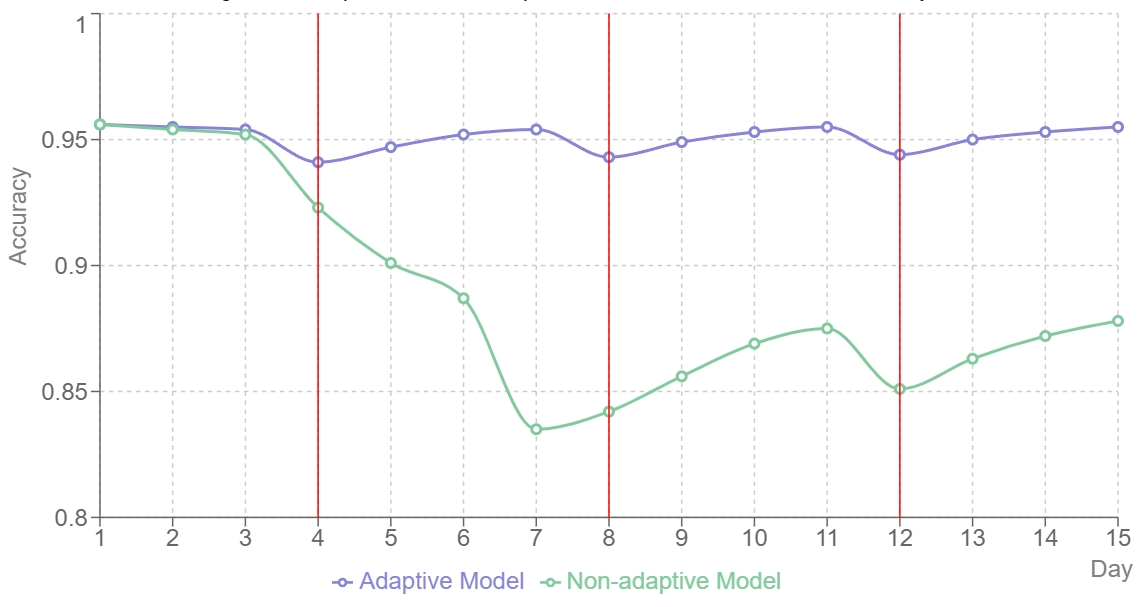

Supplement: S4 Fig — (TIF) [file pone.0332502.s004.tif]
